# Designing and implementing a training program on surgical hand scrubbing, wearing surgical cap and surgical mask, gowning, and gloving using HMD-based virtual reality technologies for nursing students: an exploration of student perceptions

**DOI:** 10.3389/fmed.2024.1364465

**Published:** 2024-06-12

**Authors:** Songül Güngör, Ayla Yava, Aynur Koyuncu

**Affiliations:** ^1^Department of Nursing, Osmaniye Korkut Ata University, Osmaniye, Türkiye; ^2^Department of Nursing, Hasan Kalyoncu University, Gaziantep, Türkiye

**Keywords:** surgical hand preparation, nursing – education, gowning and gloving, virtual reality, wearing a mask

## Abstract

**Objective:**

The aim of this study is to determine the steps of a training program utilizing Head-Mounted Display (HMD) based Virtual Reality Technology to enhance nursing students’ skills in surgical hand scrubbing, wearing surgical cap and surgical mask, gowning and gloving, and to evaluate students’ perceptions toward the program.

**Methods:**

The study aimed to investigate the potential applications of HMD-Based Virtual Reality Technology in Surgical Hand Scrubbing, Wearing Surgical Cap and Surgical Mask, Gowning and Gloving Program for nursing students, as well as students’ perceptions toward this technology. The research was conducted with a focus group consisting of second-year nursing students in Osmaniye/Turkey, between January and June 2022, and the training program was implemented in five stages: Analysis, Design, Development, Implementation, and Evaluation. The program was evaluated with a focus group of nursing students. Focus group discussions were conducted to provide insights into students’ experiences, feedback, and perceptions of the program.

**Results:**

A vast majority of participants (92.5%) reported feeling fully immersed in the operating room environment during the virtual reality (VR) experience. Notably, all students acknowledged the potential of HMD-Based Virtual Reality Technology to enrich their understanding of surgical hand scrubbing, wearing surgical cap and surgical mask, gowning and gloving procedures, surpassing conventional instructional models. While many participants found the experience exhilarating (85.1%), a considerable portion reported a decline in engagement after repeated exposures (88.8%). Overall, participants welcomed the integration of VR technology into education, expressing optimism about its capacity to facilitate additional instructional modules (74.4%). Moreover, they conveyed satisfaction with the opportunity to engage with the VR application, emphasizing its significant educational value (81.4%).

**Conclusion:**

Based on these findings, we can suggest that virtual reality technology has the potential to have an impact on nursing students’ education. The majority of students expressing a sense of presence in the operating room highlights the value of this method in education. However, the reported boredom after repeated experiences by most participants underscores the importance of diversifying the program and introducing innovative approaches to keep students engaged.

## 1 Background

Nursing is a profession that plays a critical role in the preservation, promotion, and treatment of health (1–3). The foundation of nurses’ acquisition of scientific and practical competence lies in the education they receive during their training period. The experiences gained and the confidence acquired by nursing students in classroom activities strengthen their professional identity, laying the groundwork for their future professional skills. Nursing education encompasses both theoretical and practical nursing specialty courses, including basic courses in health sciences as well as nursing science, philosophy, and practice (4, 5). One of these specialty courses, surgical nursing, aims to impart knowledge and skills in nursing care during the perioperative process of surgical interventions, whether acute or chronic diseases or trauma-related. In this context, the acquisition of important skills such as surgical hand scrubbing, wearing surgical cap and surgical mask, gowning and gloving by students is of great importance because these skills support critical objectives such as ensuring patient safety, preventing surgical site infections, and meeting quality assurance standards (1–3). The educational process for skill acquisition should consist of three stages: (1) Explanation of the theoretical aspects of the subject using visual materials, (2) Application in a laboratory setting for practical experience, and (3) Repetition steps until mastery level of the skills is achieved. However, recent research has highlighted the effectiveness of technology-based and innovative applied teaching models in improving learning outcomes in this field. Traditional teaching methods involving materials such as slide shows, pictures, graphics, or printed documents have been reported to be inadequate in helping students grasp the subject matter (10–12). On the other hand, education using Virtual Reality (VR), a new technology, has been shown to accelerate the learning process compared to traditional educational tools and facilitate long-term retention of learned information (12–14). Therefore, it is considered a more effective model in nursing education, especially as an alternative option for teaching topics that students may not experience in clinical practice due to limited clinical opportunities. VR technology can enhance the learning process by presenting real-life scenarios to students in a closer and more detailed manner (12, 15, 16). Therefore, in an application consisting of sequential and interrelated steps such as surgical hand scrubbing, wearing surgical cap and surgical mask, gowning and gloving, VR has the potential to demonstrate procedures step by step and make complex situations more understandable (17, 18). Thus, nursing students can approach surgical procedures in clinical settings more prepared and confidently. Moreover, by enhancing the quality and effectiveness of education in surgical nursing, it reduces the risks associated with errors in patient care (12, 19, 20). Therefore, this study aims to utilize HMD-Based Virtual Reality Technology as an alternative to traditional methods for teaching critical nursing skills such as surgical hand scrubbing, wearing surgical cap and surgical mask, gowning and gloving to nursing students. The development of the program and the evaluation of student feedback are important steps to understand how effective VR technology can be in surgical nursing education.

The objective of determining the program steps is to provide nursing students with an effective learning experience through virtual reality technology and to support the acquisition of surgical skills. Consequently, nursing students can be better prepared for clinical practice, enhance the quality of healthcare services, and improve patient safety. Additionally, not only nursing students but also operating room personnel and nursing educators can utilize the program. The starting point of the study is a recommendation by the Association of Perioperative Registered Nurses, emphasizing that VR simulations facilitate the recall of critical skills in clinical education, enable nurses to perform effectively with increased confidence, and positively impact patient outcomes (21). Additionally, the limited acceptance of nursing students into operating rooms (22) and their inability to acquire sufficient skills in preparing for the intraoperative process have raised the need to address this issue through virtual reality methods. Accordingly, our main goal is to provide nursing students with education on surgical hand scrubbing, wearing surgical cap and surgical mask, gowning and gloving and to evaluate their thoughts on the training. Challenges encountered during the development of VR-based training programs are often underestimated or overlooked. Particularly, identifying challenges such as inadequate preparatory work, workflow issues, and technical limitations can contribute to developing appropriate solutions to enhance the effectiveness of VR training programs (23). In the section at the end of the article, the challenges encountered during the video preparation process for the VR-based training program are identified, and strategies for overcoming these challenges are shared to prompt researchers interested in integrating technology-based education into curricula in the future. Consequently, it is expected that the development of virtual reality-based education strategies will guide nursing educators in the education of nursing students.

## 2 Methods

This study was conducted with students enrolled in the Nursing Department of the Faculty of Health Sciences at Osmaniye Korkut Ata University in Turkey during the Spring Semester of the 2021–2022 Academic Year. The study consists of two main sections. The first section focuses on the process of determining the steps of the surgical hand scrubbing, wearing surgical cap and surgical mask, gowning and gloving training program using HMD-based virtual reality technology for nursing students. The second section involves the implementation of the program and the determination of student opinions. The study was approved by the university’s ethics committee (2022/6/4), and written permission was obtained from the hospital management where the video was recorded. Consent from the participating students was obtained through three methods: electronic, written, and verbal, and a total of 27 students participated in the research.

### 2.1 Participants

The inclusion criteria for the study:

- Actively enrolled in the Nursing Department of the Faculty of Health Sciences at Osmaniye Korkut Ata University during the Spring Semester of the 2021–2022 academic year.
- Not having previous experience with VR technology in nursing education,
- First-time registration for the course in surgical nursing.
- Absence of claustrophobia and no head or neck conditions that would hinder the wearing of virtual reality goggles.

The exclusion criteria for the study:

- Graduation from a Health Vocational High School.
- Graduation from a Health Services Vocational School at the associate degree level.
- Previous participation in any webinar, congress, or meeting related to surgical hand scrubbing, wearing surgical cap and surgical mask, gowning and gloving.

The withdrawal criteria from the study:

- Non-participation in the theoretical training sessions and demonstration activities provided as part of the research.
- Expressing a desire to withdraw from the study at any stage after volunteering to participate.

### 2.2 The process of developing the surgical hand scrubbing, wearing surgical cap and mask, gowning, and gloving training program using HMD-based virtual reality technologies for nursing students

**Figure 1:**
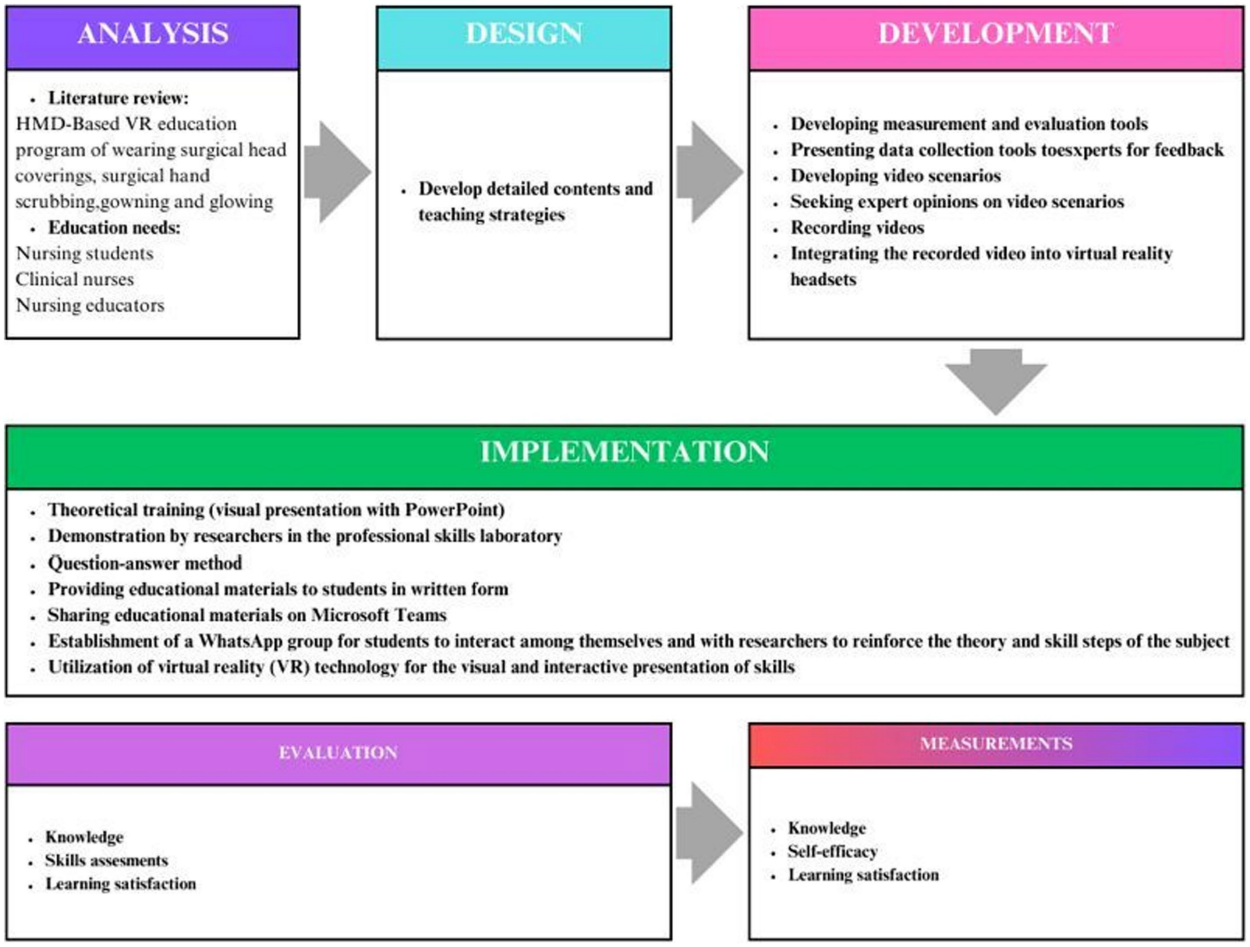
The process of developing program.

In this research, the Analysis, Design, Development, Implementation, and Evaluation (ADDIE) (Figure 1) model was utilized to create a draft of a scenario-based surgical hand scrubbing, wearing surgical cap and mask, gowning and gloving training program (24, 25). Education materials were designed to align with visual, auditory, reading/writing, and kinesthetic learning styles, and a video creation guide for nursing was employed (26, 27).

#### 2.2.1 Analysis

The analysis phase involved collecting the necessary foundational data to enhance skills in surgical hand scrubbing and sterile gloving. To achieve this, discussions were held with three expert nurses with operating room experience, two professors, and undergraduate students enrolled in the second year of the surgical nursing course (28). This helped identify the educational needs. A literature review was conducted to explore how VR addresses various learning styles in nursing education and to examine research findings (26, 27, 29–31).

#### 2.2.2 Design

In this phase of the study, our aim was to systematically classify, define, and determine the relationships among the relevant concepts. Among the parameters extensively examined within the conceptual framework of the educational program are the theoretical underpinnings of the education, the target audience of the program, the methods for providing theoretical knowledge to this audience (type of education), the methods to be used to deliver the education, and the intended outcomes of the program. The fundamental characteristics of this education program include being technology-based, serving a specific application purpose, and focusing on enhancing knowledge and skills in surgical hand scrubbing, wearing surgical cap and mask, gowning and gloving. The necessary theoretical knowledge to form the conceptual framework of the education program was compiled by the researchers. The target audience, educational method, and parameters to be developed were determined based on data obtained from the literature. This study was designed by integrating feasible theory and research findings with the aim of enhancing knowledge and skills in surgical hand scrubbing, wearing surgical cap and mask, gowning and gloving topics.

The aim of the education: to teach surgical hand scrubbing, wearing surgical cap and mask, gowning and gloving skills to second-year undergraduate nursing students.

- Determination of the target audience: Second-year undergraduate nursing students.

Theoretical framework:

- Basic principles of surgical hand scrubbing, wearing surgical cap and mask, gowning and gloving
- Infection control
- Patient safety
- Type of Training:
  - Both theoretical course content and practical skill development exercises
  - VR-supported instruction
- Teaching methods:
  - Simulations
  - Visual materials
  - One-on-one practical applications in the laboratory setting
  - Virtual reality technology
- Training content
  - Surgical hand scrubbing procedures,
  - Wearing surgical cap procedures,
  - Wearing surgical mask procedures,
  - Gowning procedures,
  - Gloving procedures,
- Principles of behavior in a sterile environment
- Target outcomes:
  - Increase in knowledge level regarding surgical hand scrubbing, wearing surgical cap and mask, gowning and gloving among students/new personnel starting to work in the operating room/newly appointed nursing educators
  - Effective application of surgical hand scrubbing, wearing surgical cap and mask gowning and gloving skills by students/new personnel starting to work in the operating room/newly appointed nursing educators
  - Understanding and implementation of best practices in infection control (32–34).

#### 2.2.3 Development

The development phase of the research consists of five steps: (1) creating measurement and evaluation tools, (2) presenting data collection tools to expert opinion, (3) developing video scenarios, (4) obtaining expert opinions for video scenarios, (5) video shooting, (6) integrating the recorded video into virtual reality headsets (35–37).

1. Creating measurement and evaluation tools: Form for the Evaluation of Skills in Donning and knowledge assessment the surgical cap and mask, surgical hand scrubbing, sterile gowning, and gloving: The form was prepared by the researcher to assess students’ skill levels in donning and knowledge assessment the surgical cap and mask, surgical hand scrubbing, sterile gowning, and gloving, in accordance with the Turkish Association of Surgical and Operating Room Nurses’ Operating Room Nursing Book’s Surgical Hand Hygiene section (2022), the Ministry of Health’s Hand Scrubbing and Hand Disinfection Guide for Health Personnel, AORN and WHO surgical hand scrubbing principles, current surgical nursing undergraduate textbooks [(38); AORN (39–49)], and expert opinions. Skills were scored on a scale of 0 to 2 for each procedural step: 0 points were assessed as “Incompetent,” 1 point as “Partially Competent,” and 2 points as “Competent.” In the skill assessment form, minimum and maximum scores that could be obtained for different skills were defined; for example, for Social Hand Scrubbing, the range was 0–2, and for Donning the Mask, it was 0–10. Overall, in this evaluation where a student could receive a minimum of 0 and a maximum of 78 points, a score of at least 55 out of 78 was required to be deemed successful (based on the Ministry of Health’s Operating Room Nursing Certification standards, which mandate that a student must achieve at least 70% in the assessment to be considered successful) (50). The knowledge assessment form consists of 10 multiple-choice questions, each with a single correct answer, covering topics such as the purpose and duration of surgical hand scrubbing, nail length and use of nail polish, preoperative preparation, donning of surgical cap and mask, availability of alternative methods to surgical hand scrubbing, and considerations for gowning and gloving. The scoring of this form, evaluated by the researcher, was based on a total of 10 points, with each question worth 1 point. The incorrect answers were not deducted from the total score, and the scoring was designed to assess the student’s knowledge level based on the number of correct answers. Higher scores indicate a higher level of student knowledge (28).
2. Presenting data collection tools to expert opinion: In the second step, the appropriateness of the questions and skill levels, and the Content Validity Index (CVI) study were conducted according to the Davis technique with the participation of five expert faculty members in the field of Surgical Nursing (51). In the Davis Technique, experts were asked to mark each question on the forms as (a) “Appropriate,” (b) “Item needs slight revision,” (c) “Item needs serious revision,” and (d) “Item not appropriate.” According to the technique, the CVI was calculated by dividing the number of experts who marked “Appropriate” and “Item needs slight revision” options (a + b) for each item by the total number of experts providing opinions, with the expectation that the CVI for each item would be 0.80 or higher. An average score of 1 for each item indicates that the item can be implemented without modification, while an item scoring 0.80 suggests that no changes are necessary, although researchers are advised to review it. Accordingly, recommendations provided by the experts were examined, and the data collection forms were reviewed and revised as necessary. The number of experts marking the “Appropriate” or “Item needs slight revision” option was divided by the total number of experts consulted, and the CVI for each question was calculated. Items scoring 0.8 or higher on the forms were reviewed by the researchers and adjusted according to the recommendations of the relevant experts. Furthermore, fifteen final-year nursing students were asked to evaluate the clarity and comprehensibility of the steps in the created form, focusing on the comprehensibility of the form. To determine the time allotted for completing the knowledge assessment form, a pilot study was conducted with 15 nursing students. In the pilot study, the response time for the test was set at 12 min.
3. Developing video scenarios: In this step, feedback was obtained via email from three faculty members specializing in surgical nursing and two nurses with doctoral education who have experience in the operating room setting, concerning the video scenario. The scenario aimed to teach surgical handwashing techniques using nail brushes and sponges, both with and without water assistance. Following the expert feedback, the scenario was finalized.
4. Obtaining expert opinions for video scenarios: In this step, feedback was obtained via email from three faculty members specializing in surgical nursing and two nurses with doctoral education who have experience in the operating room setting, concerning the video scenario. The scenario aimed to teach surgical handwashing techniques using nail brushes and sponges, both with and without water assistance. Following the expert feedback, the scenario was finalized.
5. Video shooting: The video recordings were conducted in an operating room of a hospital, with one of the researchers taking part in the video. To facilitate the stages of video shooting, a video preparation template, consisting of two parts prepared by Fleming et al. (25), was utilized. The first part detailed what the viewer would see in the video, while the second part specified features such as narration, dialog, sound effects, and background music. During the pre-production phase, which is the initial step of video creation, the previously shaped video scenario, based on expert opinions, was divided into stages using the video preparation template/shooting plan in line with the literature (25). Prior to shooting, the operating room environment where the filming would take place was inspected with the assistance of the responsible operating room technician. It was determined that the most suitable hours for filming were between 20:00–23:00 when there were no emergency surgery patients. Contact numbers of the responsible operating room technicians were obtained, their duty days were ascertained, and it was communicated that the appropriateness of the operating room would be confirmed before the filming date. Furthermore, it was emphasized that the on-call surgeon would also be informed in advance. The hospital also requested that the security unit supervisors be informed on the evenings when the filming was conducted. During the filming, it was clarified that a cameraman and equipment would accompany the researcher. Additionally, while shooting the scene of sterile dressing, the role of the circulating nurse in preparing the operating table and tying the surgical gown was proposed to the technician of the operating room where the filming would take place. This role was accepted by the respective technician. During the production phase, the researcher, in the operating room designated for the filming of surgical handwashing in the scenario, arranged the necessary materials for setting the lights and camera angles in the section where the handwashing basin was located, as well as the operating room area for sterile dressing. After conducting trial videos, it was determined that additional light sources were required to correct filming errors and enhance image quality. Consequently, additional lighting equipment was purchased. While filming, the researcher realized that they sometimes forgot certain sentences from the script and became flustered due to nervousness. To address this, after donning the bonnet, they decided to wear wireless earphones in a manner that would not be visible on the video. Accordingly, wireless earphones were acquired. The researcher recorded the voice-over for the video script on their mobile phone. By listening to this voice recording through the wireless earphones during the shoot, they planned to use it as a prompter, eliminating the need for a prompter and ensuring adherence to the script. To maintain and increase student engagement in educational videos, it is essential to keep the duration of the video concise. Therefore, preparing long videos may result in expending unnecessary energy and effort, potentially laboring in vain (25, 52). Thus, creating an 11-min video. Consequently, during the production phase, considering this assessment, the researcher practiced the tasks in the operating room to efficiently perform the role in a streamlined manner. The post-production phase, like other stages of the video production, was also undertaken by the researchers.
6. Integrating the recorded video into virtual reality headsets: In this step, the video was transferred to the Oculus Quest2 Advanced All-in-One Virtual Reality Headset (256GB). The completed video was evaluated for sound and image quality by two academics experienced in the surgical field, and an academic and a clinician, all using virtual reality headsets. The evaluators did not provide additional recommendations after reviewing the video.

#### 2.2.4 Implementation

Theoretical education (Visual presentation prepared using PowerPoint software) Demonstration by researchers in the professional skills laboratory.

Question-answer method.

Provision of educational materials in written form to students.

Sharing of educational materials on Microsoft Teams.

Establishment of a WhatsApp group for students to interact with each other and researchers to reinforce theoretical concepts and skill steps.

Utilization of virtual reality (VR) technology for the visual and interactive presentation of skills (53–58).

#### 2.2.5 Evaluation

First, the student information form was administered. Following this, a 30-min theoretical training session on gloving, including its definition, importance, and application steps, was conducted by the researcher using PowerPoint slides with visual content prepared for surgical hand scrubbing, wearing surgical cap and mask, gowning and gloving topics in one of the lecture halls of the relevant faculty. During this session, teaching methods such as question-answer sessions, discussions, and PowerPoint presentations were utilized. Subsequently, in the professional skills laboratory, the researchers provided both a recap of the theoretical knowledge of the topic and a practical demonstration of surgical hand scrubbing, wearing surgical cap and mask gowning and gloving procedures. The materials used during the theoretical training, including the PowerPoint presentation content, skill assessment steps, and written script of the video scenario, were shared with all students in the group. Additionally, the educational materials were shared on Microsoft Teams. A WhatsApp group was created to facilitate communication among students and with the researchers, allowing them to collaborate, communicate, and pose questions about the topic and skills they did not understand.

#### 2.2.6 Measurement

The virtual reality (VR) training with VR goggles was conducted once a week for a total of three weeks. Students were provided with caps, masks, sterile gloves, and surgical gowns to practice the skills in the laboratory until the knowledge and skill assessment. At the end of this process, students were asked the following questions about their training experience:

1. Did you feel like you were in an operating room during the virtual reality experience? (Yes/No)
2. How do you think HMD-Based Virtual Reality Technology affected your surgical hand scrubbing, wearing surgical cap and mask gowning and gloving learning experience? (Helped learning better/Did not help learning better)
3. Did you find the experience exciting? (Yes/No)
4. Did you feel bored after repeated experiences? (Yes/No)
5. How do you evaluate the use of virtual reality technology in education? (Positive/Negative)
6. Are you satisfied with being able to try the VR application? (Yes/No)

At the end of the three-week period, students are asked to complete the surgical hand scrubbing, wearing surgical cap and mask, gowning and gloving knowledge form in a classroom designated by the faculty. Students are individually taken to the professional skills laboratory to perform surgical hand scrubbing, wearing surgical cap and mask, gowning and gloving skills practically, while video recordings are made during this process. Meanwhile, a researcher evaluates the students using the skill assessment form that was previously validated for scope validity. Videos of students demonstrating the skills are watched by three faculty members outside of the research team and evaluated using the same skill assessment form. Subsequently, the Intraclass Correlation Coefficient (ICC) is calculated to assess the agreement among the evaluators (59, 60).

## 3 Results

Participant demographics and thoughts on the Surgical Hand Scrubbing, Wearing Surgical Cap and Mask, Gowning, and Gloving Training Program using the HMD-Based Virtual Reality Technology are presented in Table 1.

**Table 1:** Students’ demographic characteristics and perceptions of the HMD-Based virtual reality technology and surgical hand scrubbing, wearing surgical cap and mask, gowning and gloving training program (*N* = 27).

Characteristics	n	%
*Age (years): 21.37 ± 2.17 (Lowest-highest): 21–32*		
Gender		
Female	17	63.0
Male	10	37.0
It felt like being in the operating room, very realistic.	25	92.5
It was exciting.	23	85.1
It was a pleasant experience.	23	85.1
You’re not there, but you feel like you are there; it’s an impressive technology.	22	81.4
Learning through this method is a great opportunity.	22	81.4
I wish it were used in all classes.	20	74.0
It enhances learning surgical hand scrubbing, wearing surgical cap and mask, gowning and gloving.	27	100.0
It helps learning better than traditional models.	27	100.0
I got bored after experiencing it for the third time.	24	88.8

## 4 Discussion

This study aims to provide guidance for the organization and implementation of a Virtual Reality-Based Surgical Hand Scrubbing, Wearing Surgical Cap and Mask, Gowning and Gloving Program designed for nursing students. Virtual reality technology is increasingly gaining attention and becoming more widespread in nursing education. It has been suggested as an option to teach topics that students do not experience in clinical practice due to limited clinical practical time and restricted clinical settings (12, 15, 16).

Virtual reality (VR) is a technology that opens the door to a significant evolution in nursing education. Its repeatability and standardization provide a unique contribution to clinical skills training. Nursing students demand the use of educational technologies to translate the theoretical knowledge they learn about surgical diseases into practical applications in the field of nursing (23). Important organizations such as the World Health Organization and the National Council of State Boards of Nursing in the United States recommend the use of electronic learning methods in nursing school programs (61).

Farra et al. (62) and Vrillon et al. (63) emphasized that students were highly satisfied with VR-based education in their studies (62, 63). Chang (64) stated that education conducted using VR provided students with an enjoyable learning experience, and students were satisfied with this method (64). However, a meta-analysis suggested that the effect of VR education on satisfaction is not clear, and further research is needed (54). Padilha et al. (65) demonstrated that virtual simulation increased student satisfaction. In studies conducted by Lange et al. (66), Liaw et al. (67), and Saab et al. (68), nursing students stated that virtual reality is a new, simple, enjoyable, immersive, engaging, and more memorable method of learning compared to traditional educational methods (65–68). Consistent with the literature, in our study, students positively welcomed the use of virtual reality technology in education. They reported that this method could be beneficial in implementing more courses, they were satisfied with VR-based education, and they found it more effective than traditional teaching methods. VR technologies used for educational videos abstract the viewer more from external stimuli compared to other web-based methods, thereby allowing the viewer to focus more on the content. The literature indicates that VR has positive effects on both episodic and semantic memory, aiding students in retaining information for longer durations (69–71). Various research has indicated that VR positively contributes to the knowledge level of nursing students in the care of respiratory diseases transmitted via the respiratory tract in COVID-19 scenarios, chemotherapy port placement surgeries, and the preparation of surgical sets (20, 69, 72). Studies investigating the effects of VR-based education in nursing education on topics such as nursing care for patients with postoperative complications, inadvertent perioperative hypothermia, care for day surgery patients, postoperative nursing care for organs and systems, transplant surgery, compartment syndrome, amputation surgery, and nursing care are recommended for nursing researchers interested in the VR topic. Thus, specific VR training program contents can be organized for surgical nursing education on specific topics.

The majority of students in the study reported getting bored with the virtual reality application after experiencing it three times. We believe that this significant finding provides valuable insight for future research. Although it is recommended that today’s nursing students, as members of Generation Z, be trained with innovative teaching styles (73, 74), in future studies, the frequency and variety of experiences should be considered to maintain students’ attention and interest.

In conclusion, our study reflects the potential of VR as an innovative tool in nursing education and the perspectives of students after experiencing this educational method. While not completely replacing traditional teaching methods, VR can certainly complement face-to-face nursing education in skills laboratories and provide unique learning opportunities. VR offers various advantages such as immersion, interactivity, and visualization compared to traditional teaching methods. As technology continues to evolve, we anticipate that VR will play an increasingly important role in nursing education. Future research should focus on identifying specific areas where virtual reality could provide potential benefits and investigate the long-term effects of VR-based learning on knowledge retention and clinical skill development.

### 4.1 The experience of overcoming challenges

During the course of this study, we encountered several significant challenges, including securing funding for scenario filming, addressing filming errors, and ensuring operating room suitability. These challenges encompassed a range of unforeseen circumstances from the planning stage to post-filming processes. The measures and solutions taken to overcome these challenges encompass valuable lessons learned that contributed to the successful completion of the project. This section of the article aims to provide practical insights to those involved in similar projects, facing similar challenges, or striving to manage such processes more effectively.

1. Funding Challenges: We encountered difficulties in securing funding from various sources to cover the project’s budget and acquire the necessary equipment. This negatively impacted the progress of the study and delayed the start of filming as planned. Researchers personally covered the expenses incurred for the research due to the inability to secure any funding sources. Securing funding can be considered a challenge in integrating technology into education, particularly in developing countries. Our recommendations for researchers conducting future work in this area are as follows:
  - Review the study budget to reduce unnecessary expenditures and prioritize critical areas.
  - Maintain flexibility in the budget to be prepared for unexpected financial challenges.
  - Explore alternative sources of funding.
  - Increase the number of individuals in the project team to distribute expenses among more people.
  - Seek potential funding sources such as university or grant programs, local organizations, NGOs, industry connections, or establish contact with researchers and organizations abroad.
2. Dealing with Filming Errors: Technical errors such as lighting, sound recording, and camera settings during filming made the filming process more complex. Our recommendations for researchers conducting future work in this area are as follows:
  - Increase the number of trial shoots, prepare your plan as comprehensively as possible, and review each stage in advance.
  - Conduct trial shoots before filming to test the technical equipment skills of team members. These trials can help identify errors that may occur during actual filming.
  - Appoint a guide or supervisor during filming to better manage the process. This person can check technical details, make adjustments as needed, and provide guidance to team members to quickly resolve issues.
  - Learn from the errors and difficulties encountered during the filming process and continually focus on improvement. Analyze the reasons for the errors and take measures to prevent similar issues in the future.
  - Ensure you have backup plans in case of any technical errors. For example, have backup cameras or microphones available to be prepared for potential malfunctions.
  - Have a plan for emergencies at the filming location; however, it requires having a sufficient budget.
3. Operating Room Suitability Issues: The planned dates and times for video recording were postponed due to emergency surgeries, resulting in an extension of the research process. Our recommendations for researchers conducting future work in this area are as follows:
  - If conducting video recordings in a specialized area like an operating room where emergency cases may arise, spread out the filming dates over a wider time frame, taking potential emergencies and surgeries into account.
  - Set backup filming dates and be prepared to conduct the recordings on those dates if needed.
  - Complete the video recordings during academic breaks or holidays to ensure that the educational material is ready when students return to school.

## Data availability statement

The original contributions presented in the study are included in the article/supplementary material, further inquiries can be directed to the corresponding authors.

## Ethics statement

The studies involving humans were approved by Osmaniye Korkut Ata University Ethics Committee. The studies were conducted in accordance with the local legislation and institutional requirements. The participants provided their written informed consent to participate in this study.

## Author contributions

SG: Conceptualization, Methodology, Investigation, Software, Resources, Data curation, Supervision, Project administration, Visualization, Writing – original draft, Writing – review & editing. AY: Conceptualization, Methodology, Investigation, Software, Resources, Data curation, Visualization, Writing – review & editing. AK: Conceptualization, Methodology, Formal analysis, Investigation, Writing – review & editing.
